# The Protective Role of Oleuropein Aglycone against Pesticide-Induced Toxicity in a Human Keratinocytes Cell Model

**DOI:** 10.3390/ijms241914553

**Published:** 2023-09-26

**Authors:** Manuela Leri, Marzia Vasarri, Emanuela Barletta, Nicola Schiavone, Maria Camilla Bergonzi, Monica Bucciantini, Donatella Degl’Innocenti

**Affiliations:** 1 Department of Experimental and Clinical Biomedical Sciences, University of Florence, Viale Morgagni 50, 50134 Florence, Italy; manuela.leri@unifi.it (M.L.); marzia.vasarri@unifi.it (M.V.); emanuela.barletta@unifi.it (E.B.); nicola.schiavone@unifi.it (N.S.); monica.bucciantini@unifi.it (M.B.); 2 Department of Chemistry, University of Florence, Via U. Schiff 6, 50519 Sesto Fiorentino, Italy; mc.bergonzi@unifi.it

**Keywords:** pesticides, oxadiazon, imidacloprid, glyphosate, HaCaT cells, oleuropein aglycone

## Abstract

The extensive use of agricultural pesticides to improve crop quality and yield significantly increased the risk to the public of exposure to small but repeated doses of pesticides over time through various routes, including skin, by increasing the risk of disease outbreaks. Although much work was conducted to reduce the use of pesticides in agriculture, little attention was paid to prevention, which could reduce the toxicity of pesticide exposure by reducing its impact on human health. Extra virgin olive oil (EVOO), a major component of the Mediterranean diet, exerts numerous health-promoting properties, many of which are attributed to oleuropein aglycone (OleA), the deglycosylated form of oleuropein, which is the main polyphenolic component of EVOO. In this work, three pesticides with different physicochemical and biological properties, namely oxadiazon (OXA), imidacloprid (IMID), and glyphosate (GLYPHO), were compared in terms of metabolic activity, mitochondrial function and epigenetic modulation in an in vitro cellular model of human HaCaT keratinocytes to mimic the pathway of dermal exposure. The potential protective effect of OleA against pesticide-induced cellular toxicity was then evaluated in a cell pre-treatment condition. This study showed that sub-lethal doses of OXA and IMID reduced the metabolic activity and mitochondrial functionality of HaCaT cells by inducing oxidative stress and altering intracellular calcium flux and caused epigenetic modification by reducing histone acetylation H3 and H4. GLYPHO, on the other hand, showed no evidence of cellular toxicity at the doses tested. Pretreatment of cells with OleA was able to protect cells from the damaging effects of the pesticides OXA and IMID by maintaining metabolic activity and mitochondrial function at a controlled level and preventing acetylation reduction, particularly of histone H3. In conclusion, the bioactive properties of OleA reported here could be of great pharmaceutical and health interest, as they could be further studied to design new formulations for the prevention of toxicity from exposure to pesticide use.

## 1. Introduction

Pesticides are agricultural agents used to kill organisms that are hazardous to crop or livestock activities, lessen agricultural product loss, and increase food yield and quality. Insecticides, herbicides, and pesticides used to treat plant diseases are included in this definition [1].

The production of pesticides was expected to be 0.2 million tons in the 1950s and over 5 million tons in the 2000s. Approximately 350,000 tons of pesticides were sold in the EU-27 annually from 2011 to 2020 [2]. Only 1% of all pesticides are thought to be used effectively to control insects on target plants, though [1]. The vast amounts of pesticides that are left wind up on non-target plants and environmental media.

In agriculture, making treatments may be necessary, but it is important to be aware of the risks brought by such treatments. In fact, pesticides are designed to interfere with mechanisms common to various forms of life that underlie basic life functions such as respiration, energy production, nerve transmission, and reproduction. Therefore, the toxic action of pesticides goes far beyond just the target they are aimed at, and humans and the environment also become unintended victims. In fact, the toxicity of pesticides has detrimental effects on the ecological level and also on humans by causing pollution and adverse effects on human health [3]. 

Humans are exposed directly to pesticides in the workplace, and indirectly through environmental media, such as air, water, soil, and the food chain, which can be contaminated with pesticides. In fact, the scientific literature documents how not only acute poisoning, but also chronic exposure to small and repeated doses of pesticides leads to an increased risk of chronic degenerative diseases such as cancer, diabetes, respiratory diseases, neurodegenerative diseases, cardiovascular diseases, hypertension, obesity, reproductive disorders, male infertility, hormonal dysfunction, autoimmune diseases, and kidney failure [4].

The penetration rate of active and inert pesticide agents varies depending on a number of biological and environmental factors. The toxicity of these compounds to humans remains a severe issue, despite certain steps being suggested to lessen the negative impacts of pesticides on the environment and human health [4]. 

Different types of pesticides appear to have a similar effect, according to experimental data, causing oxidative stress in a variety of cellular and animal models [5]. Additionally, a number of several studies suggest that epigenetic pathways may be involved in the toxicity of pesticides. Environmental variables can activate a number of epigenetic mechanisms, such as DNA methylation, histone changes, and microRNA expression [6]. Endocrine disruptors, persistent organic pollutants, arsenic, various herbicides, and insecticides are among the kinds of pesticides that were linked to changes in epigenetic markers in in vitro studies on both humans and animals.

Much work was conducted on the need to reduce pesticide use, but there was little focus on prevention, which can help reduce the toxicity induced by pesticide exposure by reducing its effects on human health. 

Phytonutrients occur naturally in fruits, vegetables, whole grains, legumes, and nuts, and contribute to human health. A practical approach to protect human health from environmental contaminants is to consume a diet rich in phytonutrients because they can help reduce oxidative stress. Overall, reducing these risk factors can help prevent the development of a wide range of health conditions [7].

Extra virgin olive oil (EVOO), which was demonstrated to have numerous positive effects on human health [8], is a key component of the Mediterranean diet, which received particular attention in the last ten years. Oleuropein (Ole) makes up 80% of the overall proportion of polyphenols in oil.

**Figure 1:**
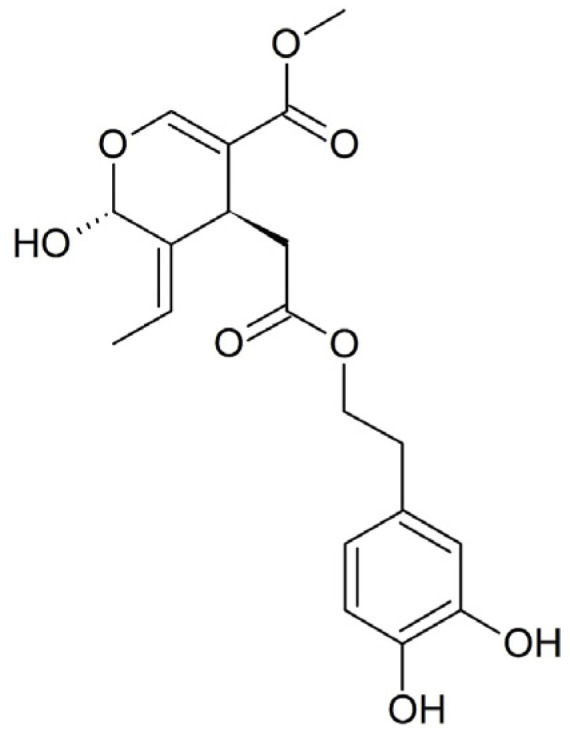
Chemical structure of oleuropein aglycone (OleA).

Scientific study primarily focuses on the aglycone form of Ole, which is an ester of hydroxytyrosol and elenolic acid that belongs to the secoiridoid glucosides group. After consumption, Ole is hydrolyzed by β-glucosidase into its corresponding aglycone (OleA), which is absorbed via passive diffusion from the gastric mucosa (Figure 1).

The OleA molecule exerts beneficial properties against numerous diseases, e.g., cardiovascular disease, diabetes, and neurodegenerative diseases through different molecular and cellular mechanisms [9,10]. Most of these effects are related to the ability of phenols to control cell signaling pathways, to modulate transcription factors activity, and to influence gene expression. Indeed, the nutrigenomic properties of EVOO and its phenolic compounds are widely reported [11], and overall, these studies explain the nutritional properties of OleA commonly associated with EVOO consumption in the Mediterranean diet.

Addressing the study of pesticide toxicity on human health, one must consider that the skin represents a possible and common route of entry for chronic exposure to small and repeated doses of these contaminants. In fact, pesticides can also be absorbed through the epidermal layers of the body [12]. However, at the same time, the skin is itself a target organ of pesticide toxicity [13].

Spontaneously immortalized non-cancerous human keratinocytes (HaCaT cells) exhibit the morphological characteristics of normal keratinocytes and express all the major surface markers and functional activities of keratinocytes. For this reason, in this preliminary work, HaCaT cells were used as an in vitro cellular model to evaluate the damaging effects of three pesticides widely used in many applications, but poorly documented in terms of sub-lethal human toxicity, and to verify the potential protective effect of OleA against cell damage. 

**Figure 2:**
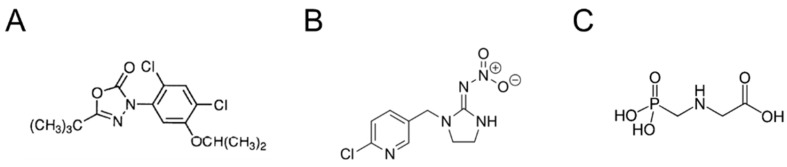
Chemical structure of (**A**) oxadiazon, (**B**) imidacloprid, and (**C**) glyphosate.

In particular, the toxicity of the herbicide oxadiazon (OXA) will be examined. OXA is a selective pre-emergence or early post-emergence herbicide belonging to the class of heterocyclic organic oxadiazole herbicides (Figure 2A). It acts on weeds with antigerminel and contact action. It is insoluble and difficult to leach. Likewise, it persists in the soil for 3–7 months. Despite its low acute toxicity, it can induce liver cancer [14], may exert adverse effects on reproduction and endocrine function [15,16], and may have neurotoxic effects with implication in the onset of neurodegenerative diseases [17].

Imidacloprid (IMID) is a systemic insecticide that belongs to the chloronicotinoid class of neonicotinoids (Figure 2B). It is an irreversible inhibitor of the nicotinic acetylcholine receptor in insects, while it is much less active on that in mammals. In recent years, IMID was reported as an emerging contaminant in all parts of the world, showing several toxic effects on a variety of nontarget organisms. IMID is generally less toxic to humans and causes mild symptoms, such as tachycardia, hypertension, and nausea; however, more severe cases were also reported, including respiratory failure, convulsions, and even death [18]. However, studies in in vivo animal models demonstrate hepatotoxic and nephrotoxic effects [19] following acute exposure to IMID, and adverse effects on male reproductive organs [20] after a sub-chronic exposure.

Glyphosate (GLYPHO) is an aminophosphorus analogue of glycine, an inhibitor of the enzyme 3-phosphoshikimate 1-carboxyvinyltransferase, known as a total non-selective herbicide (Figure 2C) [21]. It is the widest-spectrum herbicide, with an acute and chronic human toxicity of less than 94% and 90%, respectively, of all herbicides [22]. However, the toxicity of glyphosate to humans and other animals is still much debated. Today, there is much in vitro, in vivo, and epidemiological evidence on the toxicity of glyphosate in all animal species. However, because environmental exposure to glyphosate occurs from a mixture of glyphosate-based herbicides (GBH) and not from an isolated isopropylamine salt, questions remain about the environmental relevance of studying glyphosate individually. In fact, several studies show that GBH formulations are more toxic. Major hypotheses include that the GBH adjuvants are the cause of toxicity and not glyphosate itself, or that they act to increase permeation into cells, thereby increasing intracellular concentrations of glyphosate that result in toxicity, or that glyphosate and GBH adjuvants have synergistic effects [23].

In general, considering the extensive use of pesticides globally, it is increasingly important to clarify the potential toxicity induced by these molecules for human health, and to design new therapeutic approaches to prevent their adverse effects linked to their extensive use in agriculture.

In this preliminary study, the effects of the three pesticides in terms of metabolic activity, oxidative stress, mitochondrial function, and epigenetic modulation on HaCaT cells were investigated up to 24 h of treatment, and the potential protective action of OleA against pesticide-induced cell toxicity was evaluated.

## 2. Results

### 2.1. The Effect of Pesticides on the Mitochondrial Function of Human Keratinocytes

In this work, HaCaT cells were used as an in vitro cell model to evaluate the potential damaging effects of three pesticides widely used in many applications by simulating the skin as the target organ. Indeed, HaCaT cells exhibit the morphological characteristics and functional activities of normal keratinocytes.

**Figure 3:**
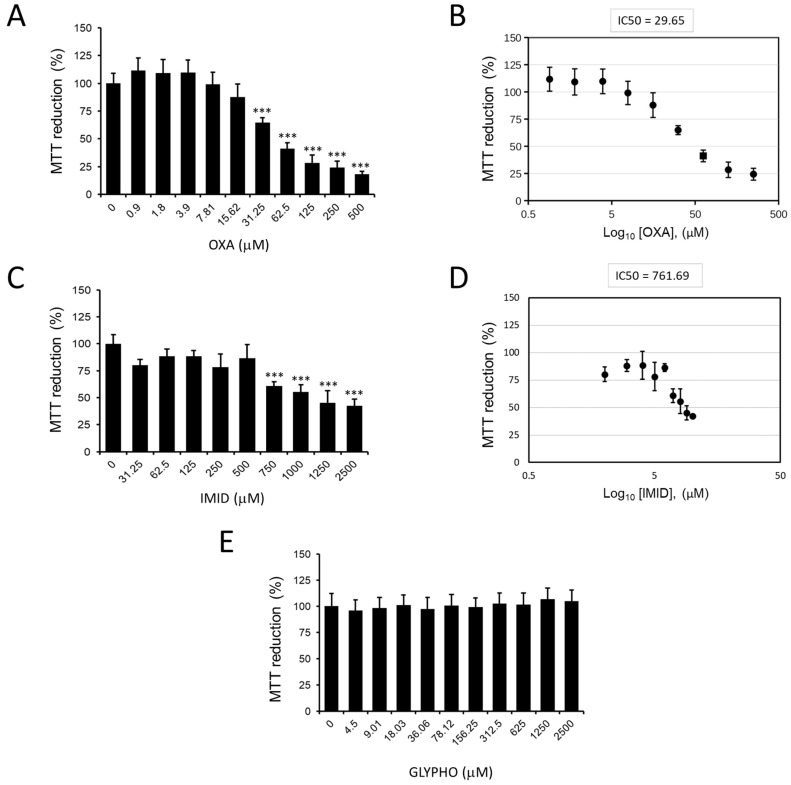
MTT assay on HaCaT cells exposed for 24 h to pesticides in serum-free DMEM medium. (**A**) Dose dependence of OXA (0–500 µM) and (**B**) calculation of the corresponding IC50 value. (**C**) Dose dependence of IMID (0–2500 µM) and (**D**) calculation of the corresponding IC50 value. (**E**) Dose dependence of GLYPHO (0–2500 µM). Values are reported as percentages compared with untreated control cells (0). Values are the mean ± standard deviation of three independent experiments. Tukey’s test: *** *p* < 0.001 vs. untreated control cells.

The MTT colorimetric assay was used to study the metabolic activity of HaCaT cells as an indicator of cell viability. Figure 3 shows the effects of the three pesticides oxadiazon (OXA), imidacloprid (IMID), and glyphosate (GLYPHO) on the viability of HaCaT cells. In particular, OXA showed dose-dependent cytotoxicity with a 50% inhibitory concentration (IC50) value of 29.65 µM (Figure 3A,B). IMID also had a dose-dependent cytotoxic effect on HaCaT cells with an IC50 value of 761.69 µM (Figure 3C,D). Different is the effect of cell treatment with GLYPHO, which caused no toxic effect on HaCaT cells at any of the doses tested (Figure 3E). Given the nontoxicity of GLYPHO at the tested doses on HaCaT cells, subsequent experiments were conducted using only the pesticides OXA and IMID.

### 2.2. The Effect of Pesticides in Terms of Oxidative Stress and Cell Death on Human Keratinocytes

Given the data obtained on the dose-dependent cytotoxicity of OXA and IMID, a potential pro-oxidant effect of these pesticides in HaCaT cells was then examined. In this regard, the production of intracellular ROS in HaCaT cells treated for 24 h with OXA (0–500 µM) and IMID (0–2500 µM) using the fluorescent probe DCFDA was evaluated. 

**Figure 4:**
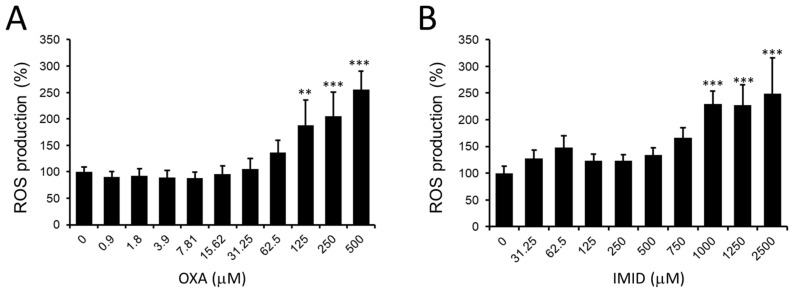
Intracellular ROS detection by DCFDA fluorescent probe on HaCaT cells exposed for 24 h to pesticides in serum-free DMEM medium. (**A**) Dose dependence of OXA (0–500 µM) and (**B**) dose dependence of IMID (0–2500 µM). Values are reported as percentages compared with untreated control cells (0). Values are the mean ± standard deviation of three independent experiments. Tukey’s test: ** *p* < 0.01, *** *p* < 0.001 vs. untreated control cells.

As depicted in Figure 4A, OXA resulted in a dose-dependent increase in intracellular ROS production in HaCaT cells from the concentration of 125 µM compared with untreated control cells; a similar effect was observed in IMID-treated cells that showed a significant increase in intracellular ROS production from the concentration of IMID 1000 µM compared with untreated control cells (Figure 4B). 

The increase in intracellular ROS induced by OXA and IMID suggests a potential involvement of the two pesticides in promoting oxidative stress in human keratinocyte cells that could result in cellular damage.

To date, there are growing concerns about the possible adverse effects of pesticide use on the population. For some time now, more attention was paid to the sustainable use of pesticides in order to reduce the risk associated with their use in various applications. Although to date exposure to the acute toxicity of pesticides is reduced due to the presence of regulations that protect workers, the related risk of sub-lethal intoxication persists, which can occur from exposures to even small amounts of the product repeated over long periods of time and is not related to the toxicity classification expressed on the label [4].

**Figure 5:**
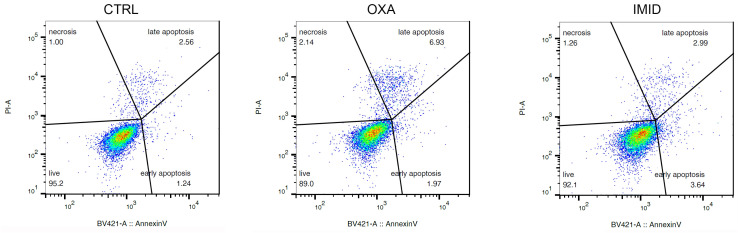
Evaluation of apoptotic process in HaCaT cells after treatment with OXA (31.25 µM) and IMID (750 µM) for 24 h in serum-free DMEM medium. The analysis conducted with FACS by staining the cells with annexin V/PI is depicted as a typical flow cytometry dot plot image. The density plot depicts the distribution of cells within a population. In these density plots, the red color represents the highest density of events within the cell population. With decreasing density, the color transitions from yellow over green to blue.

In order to “mimic” the potential damage from sub-lethal pesticide exposure on human HaCaT keratinocyte cells, sub-lethal doses of OXA (31.25 µM), and IMID (750 µM) were chosen in this work for all cell experiments below. As shown in Figure 5, the cytofluorimetric analysis performed at FACS on cells treated with OXA (31.25 µM) and IMID (750 µM) confirmed a sub-lethal effect of the two pesticides at the chosen doses showing only a slight pro-apoptotic effect.

### 2.3. Protective Effect of OleA on Pesticide-Induced Toxicity in Human Keratinocytes

In an effort to investigate possible preventive mechanisms against the adverse effects of pesticides on human health, the potential protective effect of OleA against pesticide-induced toxicity was evaluated in this preliminary work. 

**Figure 6:**
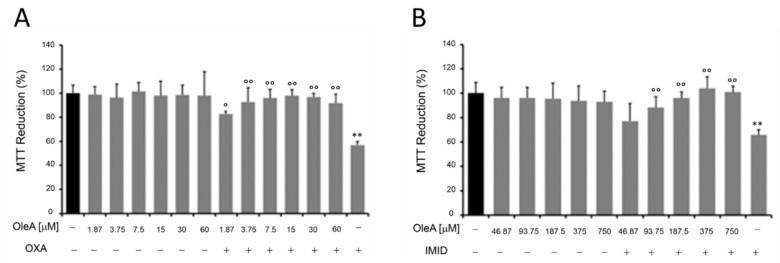
MTT assay on HaCaT cells pre-treated with OleA and then exposed to pesticides. (**A**) HaCaT cells were pre-treated with various concentrations of OleA (60, 30, 15, 7.5, 3.75, and 1.87 µM) for 24 h in serum-free DMEM medium and then exposed to OXA (31.25 µM) for an additional 24 h. (**B**) HaCaT cells were pre-treated with various concentrations of OleA (46.87, 93.75, 187.5, 375, and 750 µM) for 24 h in serum-free DMEM medium and then exposed to IMID (750 µM) for an additional 24 h. Values are reported as percentages compared with untreated control cells. Values are the mean ± standard deviation of three independent experiments. Statistics: ** *p* < 0.01 vs. untreated control cells; ° *p* < 0.05, °° *p* < 0.01 vs. pesticides-treated cells.

In this regard, HaCaT cells were pre-treated for 24 h with different concentrations of OleA and then treated with OXA (31.25 µM) and IMID (750 µM) for 24 h in serum-free DMEM medium. Cells treated only with OleA or only with pesticides were used as controls. Cell treatment with OleA did not cause any cell damage; the metabolic activity of HaCaT cells, expressed in terms of cell ability to reduce MTT, remained comparable to that of untreated control cells (Figure 6). When pre-treated with OleA, the metabolic activity of HaCaT cells was significantly enhanced compared with that of cells treated with OXA pesticide alone, while maintaining cell viability comparable to that of untreated control cells (Figure 6A). Similarly, a protective effect of OleA was also demonstrated in HaCaT cells treated with IMID. In this case, the protective effect of OleA was found to be dose-dependent (Figure 6B).

### 2.4. Protective Effect of OleA against Mitochondrial Stress Induced by Pesticide

The results obtained by MTT assay suggest that the pesticides OXA (31.25 µM) and IMID (750 µM) reduced the metabolic activity of HaCaT cells expressed in terms of cell viability by about 40%, and FACS analysis confirmed that the selected doses are sublethal since they do not induce apoptosis. For this reason, the effect of pesticides on mitochondrial membrane potential was further investigated here using the fluorescent probe MitoTracker^®^ Red. Passively diffusing through the cellular plasma membrane, the probe accumulates in active, functioning, and undamaged mitochondria, providing insights into mitochondrial dysfunction and, thus, the metabolic state of the cells.

**Figure 7:**
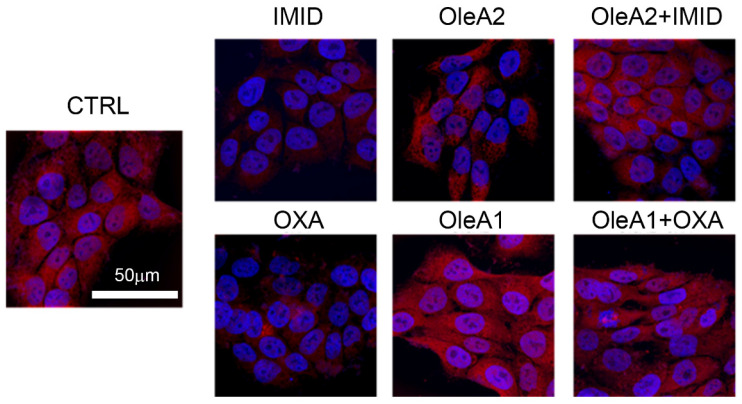
Evaluation of mitochondrial membrane potential using the MitoTracker^®^ Red fluorescent probe. HaCaT cells were pre-treated for 24 h with OleA in serum-free DMEM medium and then exposed for an additional 24 h to IMID and OXA pesticides. Specifically, the concentration of OleA 30 µM (OleA1) was used in the pretreatment of cells successively exposed to OXA (31.25 µM), while a concentration of OleA 375 µM (OleA2) was used in the pretreatment of cells successively exposed to IMID (750 µM). Nuclei were labeled with Hoechst-33342 (blue signal) and mitochondria with the MitoTracker^®^ Red probe (red signal). Scale bar = 50 µm.

As depicted by the confocal microscopy images in Figure 7, both OXA (31.25 µM) and IMID (750 µM) were able to dramatically reduce mitochondrial function within 24 h of treatment, intended as a reduction in mitochondrial membrane potential (represented by the reduction in red signal intensity). 

In view of the mitochondrial damage induced by the sublethal toxicity of the two different pesticides, the potential protective effect of OleA was evaluated. Based on the results obtained from the MTT test, the doses 30 µM of OleA (OleA1) relative to OXA treatment (31.25 µM) and 375 µM of OleA (OleA2) relative to IMID treatment (750 µM) were used. The concentration of 30 µM was chosen for OXA because it is a ratio of approximately 1:1 and is also a concentration of OleA comparable to that used to exert its beneficial activity on other cell lines [24,25]. On the other hand, for IMID, it was considered that a 1:2 OleA:IMID ratio was sufficient to achieve positive effects, also taking into consideration the bioavailability of the polyphenol itself [26]. As depicted in Figure 7, treatment with OleA alone resulted in no change in mitochondrial function; the red signal intensity was comparable to that of untreated control cells. When pre-treated for 24 h with OleA 1 and 2, cells exposed to the two pesticides showed no significant changes in mitochondrial membrane potential compared with untreated control cells (CTRL). These results suggest the efficacy of OleA in preventing mitochondrial damage induced by the sublethal toxicity of the two pesticides.

### 2.5. Protective Effect of OleA against Intracellular Ca^2+^ Fluxes Alteration Induced by Pesticide in HaCaT Cells

Calcium ions are highly versatile intracellular signals that regulate many cellular processes [27]. The damage induced by sublethal pesticide toxicity in HaCaT cells was further investigated by monitoring the variation in intracellular Ca^2+^ fluxes. It is well known that variation in intracellular Ca^2+^ fluxes can drive various cellular functions. Classically, the alteration of Ca^2+^ levels is associated with events of damage to cell membrane integrity.

**Figure 8:**
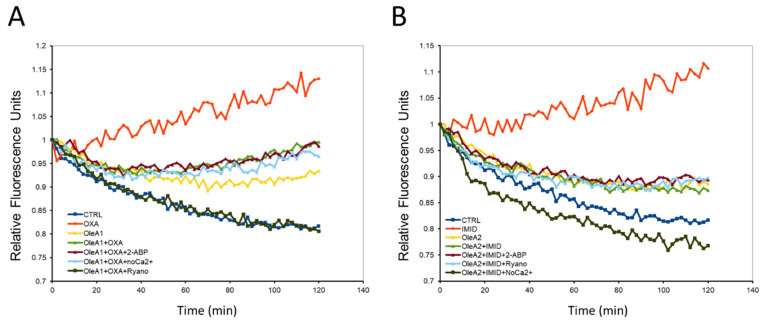
Monitoring of intracellular Ca^2+^ fluxes by fluorescent probe Fluo-3-AM. HaCaT cells were pre-treated for 24 h with OleA in serum-free DMEM medium and then exposed for an additional 24 h to OXA and IMID pesticides. Specifically, the concentration of OleA 30 µM (OleA1) was used in the pretreatment of cells successively exposed to OXA (31.25 µM) (**A**), while a concentration of OleA 375 µM (OleA2) was used in the pretreatment of cells successively exposed to IMID (750 µM) (**B**). The change in intracellular Ca^2+^ flux was measured using the fluorescent probe Fluo-3-AM. Each graph was normalized with respect to their starting point and reported with respect to untreated control cells (CTRL).

In this work, intracellular Ca^2+^ fluxes were measured in HaCaT cells using the fluorescent probe Fluo-3-AM. As depicted in Figure 8, the two pesticides OXA (31.25 µM) and IMID (750 µM) resulted in a significant increase in intracellular Ca^2+^ flux over time (0–120 min) compared with untreated control cells. Many studies showed that increased Ca^2+^ fluxes may be capable of causing cellular damage. These data thus confirm that the two pesticides OXA and IMID exert a toxic effect on HaCaT cells by causing a change in intracellular Ca^2+^ fluxes. 

The potential protective effect of OleA was then evaluated by pre-treating cells exposed to OXA (31.25 µM) and IMID (750 µM) with OleA1 (30 µM) and OleA2 (375 µM), respectively. Cell pretreatment with OleA resulted in reduced intracellular Ca^2+^ fluxes compared to cells exposed to pesticides. The literature reports that OleA is able to exert its beneficial effect by interacting with the plasma membrane. Thus, the data obtained suggest that OleA can prevent potential pesticide-induced membrane damage by interacting directly with the plasma membrane. Intracellular Ca^2+^ can originate from the extracellular space or from intracellular compartments (such as endoplasmic reticulum, lysosomes, Golgi apparatus, and mitochondria, etc.). To investigate the mechanism by which OXA and IMID pesticides were able to evoke an increase in intracellular Ca^2+^ flux, three different experimental conditions were tested: i.e., cells were treated with ryanodine (10 µM) to inhibit the release of Ca^2+^ from intracellular stores (such as the endoplasmic reticulum); cells were treated with 2-APB (100 µM), which alters the activity of nonselective ion channels on the plasma membrane; and cells were treated in a Ca^2+^-free culture medium to test whether the pesticide-induced increase in intracellular Ca^2+^ was due to Ca^2+^ entry from the extracellular compartment. When HaCaT cells are treated with the two pesticides OXA and IMID in a Ca^2+^-free medium, intracellular Ca^2+^ fluxes remain comparable to those of untreated control cells. This suggests that the two pesticides may cause an increase in intracellular Ca^2+^ flux by utilizing Ca^2+^ from the extracellular compartment. However, even when HaCaT cells were treated with the pesticides in the presence of ryanodine or 2-ABP, the pesticide-induced intracellular Ca^2+^ fluxes were significantly reduced compared with cells treated with pesticide alone. 

Taken together, these results suggest that pesticide-induced Ca^2+^ entry does not occur by a specific mechanism, but through mobilization of Ca^2+^ from multiple compartments. This effect of pesticides on intracellular Ca^2+^ flux could be due to an interaction of these highly lipophilic molecules with the plasma membrane by altering its permeability.

### 2.6. Effect of Pesticide-Induced Sublethal Toxicity on Acetylation of Histones H3 and H4, and Potential Protective Effect of OleA in Human Keratinocytes

In this work, it was shown so far that treatment with the pesticides OXA and IMID results in loss of mitochondrial function and alteration of intracellular Ca^2+^ fluxes. 

Since this early dysregulation of cellular homeostasis is often linked with epigenetic modulation, it was decided to evaluate whether sublethal concentrations of the two different pesticides had an effect on histone acetylation. 

Epigenetics is the study of heritable changes in gene expression that occur without a change in DNA sequence. Several epigenetic mechanisms, including histone modifications, can be triggered by environmental factors, such as toxins, pollutants, pesticides, and other environmental factors. There is experimental evidence indicating that epigenetic modifications can mediate the effects of these environmental factors on human health [6,28].

Post-translational modifications of histones, such as acetylation of histones H3 and H4, regulate DNA replication by affecting chromatin structures. In particular, acetylation of H4 plays a central role in regulating chromatin compaction. 

Therefore, in this work, acetylation of the two histones H3 and H4 in HaCaT cells treated with the two pesticides OXA and IMID was evaluated by confocal microscopy using primary anti-acetylation-H3 and anti-acetylation-H4 antibodies and Alexa 488-conjugated secondary antibodies (green).

**Figure 9:**
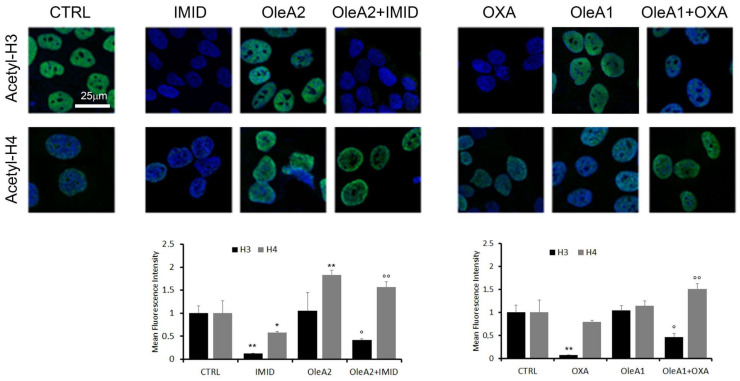
Evaluation of the acetylation status of histones H3 and H4. HaCaT cells were pre-treated for 24 h with OleA in serum-free DMEM medium and then exposed for an additional 24 h to OXA and IMID pesticides. Specifically, the concentration of OleA 30 µM (OleA1) was used in the pretreatment of cells successively exposed to OXA (31.25 µM), while a concentration of OleA 375 µM (OleA2) was used in the pretreatment of cells successively exposed to IMID (750 µM). Nuclei were labeled with Hoechst-33342 (blue signal). Acetylation was assessed using primary anti-acetylation-H3 and acetylation-H4 mouse and Alexa 488-conjugated anti-mouse secondary antibodies (green signal). Signals quantified by Fiji software were normalized to cell number and plotted in graphs. Scale bar 25 µm for all images. The mean fluorescence intensity was reported with respect to untreated control cells (CTRL). Statistics: * *p* < 0.05; ** *p* < 0.01 vs. untreated control cells (CTRL); ° *p* < 0.05; °° *p* < 0.01 vs. pesticides-treated cells.

Figure 9 shows that both IMID (750 µM) and OXA (31.25 µM) induced a significant reduction in acetylation of histone H3 of about eight times within 24 h of treatment. H4 acetylation was significantly reduced only by IMID. A dose-dependent increase in H3 and H4 acetylation was detected in OleA-treated cells, in agreement with a recent study showing that the beneficial effects of olive oil phenolic compounds, including OleA, on health are attributed to the ability of these compounds to induce epigenetic modifications such as histone modifications [11]. 

The potential protective effect of OleA against pesticide-induced epigenetic alterations was then evaluated. When cells were pre-treated for 24 h with OleA and then with pesticides, a partial suppression of the pesticides-induced effect on H3 acetylation was shown. Otherwise, the OleA pre-treatment maintained high levels of H4 acetylation in the presence of both pesticides.

Overall, this result further confirms the protective effect of OleA against the toxicity exerted by pesticides.

## 3. Discussion

Nowadays, agricultural activities rely heavily on the use of pesticides [29,30], which brought great benefits in increasing food availability and quality [31]. However, the misuse or overuse of pesticides causes a wide range of negative impacts on the environment, species diversity, and animal and human health, as is well documented by numerous toxicological studies [32]. The skin may be a major route of human exposure to pesticides, especially for the occupational population, as well as itself a target organ of exposure with adverse consequences, depending on the dose and time of exposure [12]. 

In this work, three pesticides with different physicochemical and biological properties, namely oxadiazon (OXA, herbicide), imidacloprid (IMID, insecticide), and glyphosate (GLYPHO, herbicide), were selected to evaluate their cytotoxicity in an in vitro cell model of human keratinocytes (HaCaT cells). This choice was motivated by the fact that the three pesticides have different chemical structures (Figure 1), different water solubility, and are directed at different molecular targets. 

Regarding the toxicity of OXA in in vitro and in vivo cellular models, few studies are published. In primary human striatal neuronal precursor cells (HSP cells), Barletta et al. reported that 62.50 μM was the dose of OXA corresponding to about 50% of cell viability after 24 h of exposure [17]. On the other hand, Huang et al. used a zebrafish embryo as a model to evaluate the toxicity of the herbicide oxadiazon-butachlor (OB) in aquatic organisms, and a lethal concentration 50 of OB of 9.341 mg/L at 24 h post-fertilization was calculated. It was also shown that OB affects early cardiac development in zebrafish, causing cardiac damage and cardiotoxicity by triggering oxidative stress processes and cell apoptosis [33]. 

OXA-induced toxicity was studied here for the first time in the human keratinocyte HaCaT cell line by simulating OXA skin exposure. Cell viability was analyzed by MTT assay, and an IC50 of 29.65 µM was calculated after 24 h of OXA exposure. 

Regarding the toxicity of IMID toward cell lines, Silva et al. demonstrated a reduction in cell viability in Caco-2 cells exposed for 24 h or 48 h to IMID concentrations above 200 µM [34]. Guimarães et al. showed, instead, that within 24 h of IMID exposure, in the range of 0.5–2.0 mM, the viability of HepG2 cells was dose-dependently reduced [35]. Singh et al. recently reported that IMID at 2.35 mM reduced the viability of HaCaT cells below 50% when exposed for 24 h [36]. These literature values are well aligned with the toxicity of IMID that was demonstrated in this work toward HaCaT cells; in fact, an IC50 of 761.69 µM was calculated from the MTT assay after 24 h of exposure, with values lower than 50% of cell viability, for 2.5 mM IMID concentrations. 

Regarding the toxicity of GLYPHO to some cell lines, a previous study on Caco-2 cells showed that concentrations above 59 mM disrupted Caco-2 monolayers [37]. In contrast, Mesnage et al. identified an IC50 value in Caco-2 cells of 102 mM using a GLYPHO-based formulation [38]. Regarding the effect of GLYPHO on skin cells, in a melanocyte cell line (SK-MEL-2), an IC50 of 11 µM was reported after 72 h of exposure to GLYPHO [39], while in HaCaT cells, Heu et al. reported an IC50 of 30 mM after 18 h of exposure [40]. However, the concentrations of GLYPHO used in these studies are much higher than human exposure. 

Accordingly, in this work, a range of concentrations up to 2.5 mM GLYPHO was chosen, which is closer to the population exposure reported in the literature [41]. At the selected concentrations of GLYPHO, no signs of cellular toxicity were found in HaCaT cells after 24 h of exposure in this study. Comparing the toxicity of the three pesticides, GLYPHO was found to be nontoxic, and this result can be explained by the absence of the target enzyme of GLYPHO in animal cell lines, thus it does not interfere with cell metabolism, but also because it is a highly water-soluble acid compound (water solubility of 10 g/L) with respect the other two used pesticides [42]. Therefore, we can postulate that the water solubility of GLYPHO and its negatively charged areas could reduce its ability to interact with cell membranes. 

Instead, the high liposolubility of OXA (water solubility of 0.7 mg/L) [43] and IMID (water solubility of 0.61 g/L) [44] could promote their cellular uptake and toxicity. 

Lipophilicity is a critical characteristic of xenobiotics, determining the ability to cross or be retained in the lipid bilayer barrier. At 24 h exposure to both OXA and IMID, HaCaT cells, indeed, showed a dose-dependent increase in intracellular ROS production triggering oxidative stress that could lead to cellular damage. As reported in the literature, pesticide-induced oxidative stress can be induced by an increase in lipid peroxidation and a decrease in antioxidant capacity due to their ability to interact with plasma membranes [5]. There is indeed experimental evidence showing that exposure to IMID caused significant lipid peroxidation and alteration of antioxidant enzyme activities [45,46].

To “mimic” the potential damage caused by low and sublethal pesticide exposure to human HaCaT keratinocyte cells, the toxicity mechanisms of OXA and IMID were studied using sub-lethal doses of the two pesticides. FACS analysis confirmed that the chosen doses of 31.25 µM of OXA and 750 µM of IMID were sub-lethal doses that did not cause apoptotic cell death, although they strongly reduced cellular metabolic activity and mitochondrial function, as evidenced by MTT assay and analysis of mitochondrial membrane potential by confocal microscopy. Notably, numerous studies all confirmed that the decreased mitochondrial functionality is a critical mechanism related to oxidative stress. Generally, the organochloride class, to which OXA and IMID belong, was described to impair mitochondrial function [47]. 

Alteration of calcium homeostasis is recognized as one of the mechanisms responsible for mitochondrial bioenergetic dysfunction [48]. In this work, it was observed that the two pesticides OXA and IMID resulted in increased intracellular calcium fluxes in HaCaT cells compared to untreated control cells. Li et al. showed that neonicotinoid insecticides, a class to which IMID belongs, trigger mitochondrial bioenergetic dysfunction through manipulation of the ROS-calcium pathway and increase Ca^2+^ influx through regulation of Ca^2+^ entry into the storage in an experimental mice model [49].

However, in this work, we showed that the pesticide-induced Ca^2+^ entry was not related to a specific mechanism. The high lipophilicity of OXA and IMID could target cell membranes to alter their permeability and thus promote nonspecific Ca^2+^ mobilization from multiple compartments.

Dysregulation of cellular homeostasis is often linked to epigenetic modulation [50]. Several epigenetic mechanisms, including histone modifications, can be triggered by environmental factors, such as toxins, pollutants, pesticides, and other environmental factors. There is experimental evidence indicating that epigenetic modifications can mediate the effects of these environmental factors on human health.

Changes in amino acids residing on the N-terminal tails of histone proteins can affect all DNA-based processes, including chromatin compaction, nucleosome dynamics, and transcription [51]. In this work, sublethal concentrations of OXA and IMID were shown to result in changes in the acetylation of histones H3 and H4. Experimental evidence attributed epigenetic modifications to cause the development of diseases such as leukemia [52] or neurodegenerative disorders [53] following exposure to organochlorine pesticides. Therefore, the exposure to these pesticides could underlie the onset of human diseases.

Over the years, much work was conducted on the need to reduce pesticide use, but there was little focus on prevention, which can help to reduce the toxicity induced by pesticide exposure. 

One practical approach to protect human health from damage induced by environmental contaminants is to consume a diet rich in phytonutrients. In fact, these natural molecules were long used in traditional medicine for their beneficial properties for human health and can help reduce oxidative stress and/or manage various injuries.

Overall, reducing these risk factors can help prevent the development of a wide range of unhealthy conditions.

In recent years, many studies focused on phytocompounds, bioactive molecules found in food and in dietary supplements, which are important against age-associated chronic diseases, and there is increasing evidence of the beneficial effects of the Mediterranean diet, probably due to its high polyphenol content [54]. Polyphenol content in food is rather higher than any other food ingredient with antioxidative properties. They are plant metabolites with specific properties that allow them to act as protection against UV radiation and damage caused by pathogens [55]. As was observed in this work, pesticides induce cellular damage by acting on mitochondrial function and increasing ROS production. In this context, phenolic compounds appear to be very promising molecules, considering that they mainly act as “free radical scavengers” [56,57].

In fact, researchers recently focused on the ability of polyphenols to counteract pesticide damage, and among them, phenolic compounds such as epigallocatechin gallate and curcumin were found to ameliorate pesticide-induced oxidative stress and inhibit pesticide-induced apoptosis [58,59]. Accordingly, because of their excellent antioxidant properties and strong free radical scavenging action at the cellular level, phenolic compounds were tested as potential food ingredients that can reduce the toxic effects of pesticides [60].

In this context, recent cosmeceuticals, a mix of products between cosmetics and pharmaceuticals, include some natural products, such as caffeine, capsaicin, and cyanoacrylates, which are ingredients for cosmetic blocks with broadly protective effects on the skin. Their extensively different uses are due to their action against multiple target proteins involved in the onset of several diseases. This is a recent aspect that should be considered in accordance with the recent theory of “Drug repositioning” [61]. The beneficial effects of EVOO polyphenols are not limited to their antioxidant action. In fact, leaf and olive extracts are traditionally used to treat dermatological disorders such as acne, psoriasis, rosacea, and eczema [62]. Due to their skin moisturizing power and the antioxidant and anti-inflammatory capacity of the phenolic compounds contained in EVOO, they are very interesting as additives for topical hygiene and cosmetic products [63]. Indeed, oleuropein, the main polyphenol in EVOO, resulted in a useful treatment for preventing skin photoaging due to its ability to inhibit photoinduced oxidation processes found in membrane structures [64]. Furthermore, by exploiting the polyphenolic compounds present in vegetation waters, the pharmaceutical industry can contribute to the reduction in waste generated by the oil-making process and unlock a new source of valuable and sustainable ingredients. 

This study reveals an unprecedented preventive effect of OleA against pesticide-induced toxicity in terms of mitochondrial functionality, alteration of intracellular Ca^2+^ levels, and epigenetic modifications, particularly acetylation of H3 and H4. 

All these results lead to the hypothesis that even on HaCat cells, OleA may: (i) interact directly with the cell membrane, and (ii) modulate specific intracellular pathways by acting on cell surface receptors. Indeed, because of the important hydrophobic character of OleA and its high phospholipid/water partition coefficient, some of its possible effects on biological systems could be related to its ability to interact and localize in the cell membrane [65]. Moreover, it was reported that OleA modulates several receptors to exert its beneficial health effects; for example, it acts on human epidermal growth factor receptor 2 (HER2) in breast cancer [66] or activates transient receptor ankyrin potential 1 (TRPA1) and vanilloid receptor 1 (TRPV1) to reduce visceral fat content in obese rats induced by a HF diet [67]. In addition, it was recently shown that the anti-inflammatory activity of OleA is accomplished through the activation of TREM2 on the surface of microglia cells [24].

## 4. Materials and Methods

### 4.1. Materials and Reagents

Dulbecco’s modified Eagle’s medium (DMEM), foetal bovine serum (FBS), penicillin and streptomycin, L-glutamine, trypsin-EDTA solution, phosphate-buffered saline (PBS), 1-(4,5-dimethylthiazol-2-yl)-3,5-diphenyl formazan (MTT), 2′,7′-Dichlorofluorescin diacetate (DCFDA) fluorescent probe, and other chemicals of an analytical grade were purchased from Merck KGaA (Darmstadt, DA, Germany).

MitoTracker CMXRos fluorescent dye, Hoechst 33342 nucleic acid stain, and the Fluo-3 acetoxymethyl ester (Fluo-3 AM) fluorescent probe were supplied by Thermo Fisher Scientific (Monza, Italy). 

Rabbit primary antibodies acetyl histone H4 (K5) (#9672S) and acetyl histone H3 (K9) (#9649S) were purchased from Cell Signaling Technology (CST, Danvers, MA, USA). The Alexa 488-conjugated anti-rabbit secondary antibody was purchased from Thermo Fisher Scientific, Milan, Italy). Sarstedt (Nümbrecht, Germany) provided the disposable plastic. The pesticides oxadiazon (OXA), imidacloprid (IMID) and glyphosate (GLYPHO) with purity (HPLC) ≥98% were purchased from Merck KGaA (Darmstadt, DA, Germany). IMID and OXA were resuspended in DMSO at 1 M and 125 mM, respectively. GLYPHO was resuspended in aqueous solution at 31.25 mM. 

Oleuropein was purchased from Extrasynthese (Genay, France) with purity (HPLC) ≥98%, and deglycosylated by treatment with almond β-glycosidase (EC 3.2.1.21, Fluka, Sigma-Aldrich) as previously described [68]. Briefly, a 10 mM solution of oleuropein in 310 μL of 0.1 M sodium phosphate buffer (pH 7.0) was incubated with 8.9 I.U. of β-glycosidase overnight at room temperature. The reaction mixture was centrifuged at 18,000 rpm for 10 min to precipitate. The complete oleuropein deglycosylation was confirmed by assaying the glucose released in the supernatant with the glucose (HK) assay kit (Sigma-Aldrich). Stocks of oleuropein aglycone (OleA) were kept frozen and protected from light, and were used within the same day once opened. OleA was resuspended in DMSO (dimethylsulfoxide) at 50 mM concentration.

### 4.2. Cell Line and Culture Conditions

HaCaT cells are spontaneously transformed keratinocytes from histologically normal skin, and were purchased from Cell Line Service (CLS, 300493). Cells were grown in DMEM medium supplemented with 2 mM L-glutamine, 100 µg/mL streptomycin, 100 U/mL penicillin, and 10% FBS (complete medium) at 37 °C in a 5% CO_2_-humidified atmosphere. At 80–90% confluence, the cells were detached with 0.025%-EDTA 0.5 mM trypsin solution, and propagated after appropriate dilution. 

The following in vitro cell-based experiments were performed in serum-free DMEM medium. 

### 4.3. MTT Assay

The MTT colorimetric assay was used to study the metabolic activity of HaCaT cells as an indicator of cell viability. Briefly, cells were grown in a 96-well plate (1 × 10^4^ cells/well) in complete medium overnight. To assess cell viability following pesticide exposure, HaCaT cells were treated with different concentrations of OXA (0.9–500 µM), IMID (31.25–2500 µM), or GLYPHO (4.5–2500 µM) in serum-free DMEM medium for 24 h. To evaluate the protective effect of OleA on the sub-lethal dose of the pesticides OXA and IMID, HaCaT cells were pre-treated for 24 h with different concentrations of OleA (1.87–60 µM) and then exposed for 24 h to OXA (31.25 µM), or concentrations of OleA (46.87–750 µM) and then exposed for 24 h to IMID (750 µM) in serum-free DMEM medium for 24 h. Untreated cells were used as controls (CTRL).

Subsequently, the culture medium was removed and 100 µL/well of MTT solution (0.5 mg/mL) was added. After 1 h of incubation in the dark at 37 °C, cells were lysed in 100 μL of dimethyl sulfoxide (DMSO) and absorbance values were measured at 595 nm with the iMARK microplate reader (Bio-Rad, Philadelphia, PA, USA). Data were expressed as a percentage compared with CTRL.

### 4.4. Measurement of Intracellular ROS Level

An intracellular ROS level was measured using the cell-permeable fluorescent probe 2,7-dichlorodihydrofluorescein diacetate (DCFDA). Cells were seeded in a 96-well plate (1 × 10^4^ cells/well) in complete medium and incubated overnight. To assess cell viability following pesticide exposure, HaCaT cells were treated with different concentrations of OXA (0.9 to 500 µM) and IMID (31.25 to 2500 µM) in serum-free DMEM medium for 24 h. Next, DCFDA probe (10 μM in PBS) was added to each well and incubated in the dark for 1 h at 37 °C. Fluorescence values were measured at the excitation and emission wavelengths of 485 and 538 nm, respectively, using a Biotek Synergy 1H plate reader (Agilent Technologies, Santa Clara, CA, USA). Intracellular ROS level was normalized to cell viability. Data were reported as percent compared with untreated control cells (CTRL).

### 4.5. Apoptosis Assay by FACS

Cell viability was also assessed by identifying apoptotic cells via FACS analysis using a commercially available kit (Annexin V-FITC apoptosis detection kit, #ab14085, Abcam, Cambridge, UK). HaCaT cells were seeded in 6-well plates at a density of 2 × 10^5^ cells/well. Cells were then treated with a single chosen dose of OXA (31.25 µM) and IMID (750 µM) for 24 h in serum-free DMEM medium. Untreated cells were used as control (CTRL). After treatments, cells were collected by centrifugation and resuspended in 500 μL of 1× annexin binding buffer, and incubated with 5 μL of annexin V-FITC (BV421 annexin V, BD Biosciences) and 5 μL of propidium iodide (PI) at room temperature for 5 min in the dark. Cells were analyzed with BD FACS Canto II (BD Biosciences, Franklin Lakes, NJ, USA). Data were analyzed with FlowJo software version 10.6. Cell distribution according to annexin V and/or PI positivity allowed measurement of the percentage of viable cells (annexin V and PI negative), early apoptosis (annexin V positive and PI negative) or late apoptosis (annexin V and PI positive). A minimum of 10,000 events was collected.

### 4.6. Evaluation of Mitochondrial Membrane Potential

MitoTracker Red^®^ mitochondrial fluorescent probe CMXRos was used to label the biologically active mitochondria in the cell and detect the mitochondrial membrane potential (MMP). Briefly, HaCaT cells (50 × 10^5^ cells/well) were grown on coverslips in complete medium overnight. The cells were then pre-treated with OleA for 24 h in serum-free DMEM medium and then exposed to OXA and IMID pesticides for another 24 h in serum-free DMEM medium. Specifically, cells were pre-treated with 350 µM OleA (OleA1) and then treated with IMID (750 µM), or with 30 µM OleA (OleA2) and then treated with OXA (31.25 µM). Next, cells were incubated at 37 °C for 45 min with MitoTracker CMXRos (500 nM) to detect mitochondria membrane potential, and Hoechst-33342 (10 μg/mL) for nuclei staining. Then, cells were fixed in 2% buffered paraformaldehyde for 10 min and washed twice in PBS. Cover slips were placed on microscope slides using a Fluoromount™ aqueous mounting medium (Merck KGaA, Darmstadt, DA, Germany), and images were collected using a Leica TCS SP8 scanning microscope (Leica, Mannheim, Germany) equipped with an APO HCX Plan 63×, 1.4–0.6 NA, oil objective. Images were acquired with Leica LAS-AF acquisition software (version 5.0.1). Photomontages were generated with FiJi software, version 8.

### 4.7. Measurement of Cytosolic Calcium Levels

Cytosolic levels of free Ca^2+^ within live cells were measured using the fluorescent probe Fluo-3 acetoxymethyl ester (Fluo-3 AM). HaCaT cells were seeded on sterilized slides and incubated at 37 °C for 5 min with 5 μM Fluo-3 AM. Then, the cells were pre-treated with OleA for 24 h in serum-free DMEM medium and then exposed to OXA or IMID pesticides for a further 24 h in serum-free DMEM medium. Specifically, cells were pre-treated with OleA1 (350 µM) and then treated with IMID (750 µM), or with OleA2 (30 µM) and then treated with OXA (31.25 µM). To investigate the potential mechanism by which pesticides affected intracellular calcium flux, three different experimental control conditions were tested. Specifically, cells were pre-treated for 1 h with 2-aminoethoxybiphenyl borate (2-APB, 100 µM), a reliable blocker of Ca^2+^ entry operated by deposits, or with ryanodine (10 µM), responsible for Ca^2+^ release from intracellular deposits, or in a Ca^2+^-free culture medium. 

The subsequent fluorescence can be detected by monitoring the increase in fluorescence at 526 nm for 2 h of acquisition. Fluorescence intensity of Fluo3-AM was measured using a Biotek Synergy 1H plate reader in continuum.

### 4.8. Analysis of Histone H3 and H4 Acetylation by Immunofluorescence Assay

HaCaT cells were seeded on sterilized slides in a 24-well plate at a density of 4 × 10^4^ cells/well overnight. Then, cells were pre-treated with OleA for 24 h in serum-free DMEM medium and then exposed to OXA or IMID pesticides for another 24 h in serum-free DMEM medium. Specifically, cells were pretreated with OleA1 (350 µM) and then treated with IMID (750 µM) or with OleA2 (30 µM) and then treated with OXA (31.25 µM). The cells were then treated with Hoechst-33342 (10 μg/mL) for nuclei staining for 40 min at room temperature. Next, cells were fixed in 2% (*v*/*v*) paraformaldehyde for 6 min and permeabilized with a cold 1:1 acetone/ethanol solution for 4 min at room temperature. The cells were then blocked in a 0.5% (*w*/*v*) BSA solution and 0.2% (*w*/*v*) gelatin. Acetylation of histones H3 and H4 was stained with the rabbit antibodies anti-acetyl histone H4 (K5) and anti-acetyl histone H3 (K9), used in 1:500 dilution in the blocking solution for 1 h incubation at 37 °C. After 30 min of washing in PBS under agitation, the cells were incubated with Alexa 488-conjugated anti-rabbit secondary antibody (at 1:100 dilution in PBS). Finally, the cells were washed twice with PBS and once with distilled water to remove non-specifically bound antibodies. Fluorescent signals were detected with a Leica TCS SP8 scanning confocal microscope (Leica, Mannheim, Germany) equipped with a HeNe/Ar laser source for fluorescence measurements. Observations were made with a Leica HC PL Apo CS2×63 oil immersion objective. Image analysis was performed using Fiji ImageJ software, version 8.

### 4.9. Statistical Analysis

When not specified, statistical analysis of the data was performed by using one-way analysis of variance (ANOVA).

## 5. Conclusions

This study compared the toxicity of three different pesticides in an in vitro skin cell model using human HaCaT keratinocytes. The demonstrated toxicity of sub-lethal doses of oxadiazon (OXA) and imidacloprid (IMID) in terms of reduced metabolic activity, mitochondrial function, and epigenetic modification in HaCaT cells could be attributed to their high lipophilicity and ability to interact with the plasma membrane, inducing oxidative stress and altering intracellular calcium fluxes.

In order to identify molecules capable of preventing the toxicity induced by sublethal exposure to pesticides, the potential benefits of oleuropein aglycone (OleA), already known for its innumerable beneficial properties for humans, were investigated in this work. The demonstrated ability of OleA to prevent the toxicity induced by OXA and IMID in HaCaT cells could be a good starting point to investigate the possibility of using this molecule in the formulation of skin products for preventive purposes during acute or chronic exposure to pesticide use.

Despite the interesting results obtained in this study, it should be noted that the sub-lethal effect of pesticides was tested in 24 h and a 2D cell culture model was used. However, this preliminary study provides a solid basis for further study and investigation of the effect of chronic pesticide exposure (2–3 days) in a 3-dimensional epidermal model. In conclusion, our work improves the knowledge of the molecular mechanisms of OleA and provides further useful information for its use as a therapeutic or cosmetic agent in new effective and sustainable formulations for skin protection.

## Data Availability

We decided to not share our data.
